# New *SMARCA2* mutation in a patient with Nicolaides–Baraitser syndrome and myoclonic astatic epilepsy

**DOI:** 10.1002/ajmg.a.37935

**Published:** 2016-09-26

**Authors:** S. Tang, E. Hughes, K. Lascelles, EuroEPINOMICS RES myoclonic astatic epilepsy working group, M. A. Simpson, D. K. Pal

**Affiliations:** ^1^ King's College London London United Kingdom; ^2^ King's Health Partners London United Kingdom

**Keywords:** SMARCA2, myoclonic astatic epilepsy, Nicolaides–Baraitser syndrome

## Abstract

We report a de novo *SMARCA2* missense mutation discovered on exome sequencing in a patient with myoclonic astatic epilepsy, leading to reassessment and identification of Nicolaides–Baraitser syndrome. This de novo *SMARCA2* missense mutation c.3721C>G, p.Gln1241Glu is the only reported mutation on exon 26 outside the ATPase domain of SMARCA2 to be associated with Nicolaides–Baraitser syndrome and adds to chromatin remodeling as a pathway for epileptogenesis. © 2016 The Authors. *American Journal of Medical Genetics Part A* published by Wiley Periodicals, Inc.

## INTRODUCTION

Myoclonic astatic epilepsy (MAE), or Doose syndrome, is a genetically heterogenous rare childhood epilepsy syndrome characterized by the onset of myoclonic‐atonic or atonic seizures between the ages of 6 months and 6 years in a previously normally developing child [Doose et al., 1970]. Typical febrile seizures may precede afebrile seizures in two‐thirds of cases. Other generalized seizure types are frequently seen and electroencephalogram (EEG) shows generalized spike wave or polyspike discharges on a normal background. Prognosis is variable from complete seizure remission, with normal or close to normal development, to a drug‐resistant epileptic encephalopathy. Approximately 10% of cases can be attributed to mutations in *SCN1A*, *SCN1B*, *GABRG2*, *SLC2A1*, *CHD2*, or *SLC6A1*, but the majority of cases remain unexplained [Wallace et al., 1998, 2001; Escayg et al., 2001; Mullen et al., 2011; Carvill et al., 2013; Carvill et al., 2015]. We report a patient with MAE who, following exome sequencing, was subsequently diagnosed with Nicolaides–Baraitser syndrome (NCBRS) (OMIM 601358). NCBRS was first described in 1993 and is an intellectual disability and multiple congenital anomalies syndrome associated with seizures [Nicolaides and Baraitser, 1993]. Although much of the phenotype is well delineated and includes sparse hair, microcephaly, typical facial morphology, brachydactyly, prominent interphalangeal joints, and intellectual disability with marked language impairment, the epilepsy features have not been well described before.

## CLINICAL REPORT

A girl of Ghanaian ancestry was born at term with a birth weight of 3.4 kg (50th centile) and a head circumference of 32 cm (0.4th centile). Her mother reported normal early developmental milestones. She sat unsupported at 8 months, walked at 12 months, and was babbling just before her first birthday. However, her mother had concerns that she had reduced visual interest in people and did not smile readily. Her first seizure was at 14 months and was a myoclonic atonic seizure, which continued as her prominent seizure type while she developed generalized tonic‐clonic and absence seizures. Additionally, the patient had feeding problems from 6 months following the introduction of solids. She was hypersensitive to textures and demonstrated food refusal and would hold food in her mouth or vomit during meal times. Developmental assessment showed a delay in language and social communication skills. No dysmorphic features were identified at the time. At 17 months her weight was 10.7 kg (50th–75th centile), height 82.5 cm (75th–91st centile), head circumference 48 cm (98th–99.6th centile). At 2 years 9 months, her weight was 13.9 kg (25th–50th centile), height 94.7 cm (50th–75th centile), and she had developed relative microcephaly with her head circumference at 49 cm (9th–25th centile). Her EEG demonstrated frequent generalized bursts of polyspike and wave activity during wakefulness and sleep against a normal background. An electrographic correlate of negative axial myoclonus was captured (see Supplementary information Figs. S1 and S2). An MRI brain scan was normal. Array CGH analysis with ∼44,000 probes across the genome was normal. Based on these electroclinical features, her epilepsy type was thought compatible with a diagnosis of MAE. She was treated with sodium valproate to which she responded, attaining complete seizure remission at 3 years with normalization of her EEG. Antiepileptic medication was discontinued at 4 years.

The patient was reviewed at 5 years. She had developed a restricted pattern of feeding with aversion to lumpy foods. The 3Di autism interview revealed abnormalities in communication and non‐verbal communication, with borderline scores in social reciprocity and restricted/repetitive behavior and interests, and she was diagnosed with autism spectrum disorder. On neurodevelopmental assessment with the Wechsler Preschool and Primary Scale of Intelligence III, she demonstrated severe learning difficulties and scored 51 for Verbal IQ (0.1th centile), 57 for Performance IQ (0.2th centile), and 47 for Full Scale IQ (<0.1 centile). Repeat awake and sleep EEG and repeat MRI brain scans were normal.

**Figure 1:**
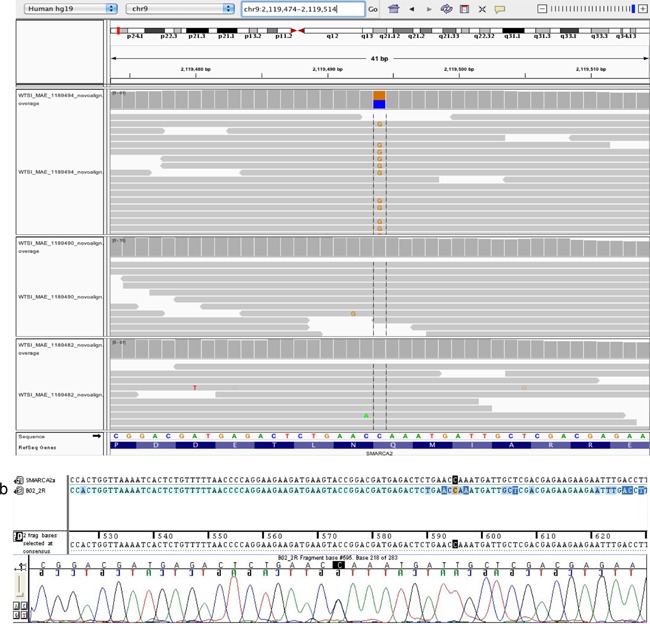
**a**: IGV plot demonstrating the de novo *SMARCA2* mutation; patient (top), mother (middle), and father (bottom). **b**: Sanger sequencing chromatogram of the patient's *SMARCA2* mutation. [Color figure can be viewed at wileyonlinelibrary.com].

Exome sequencing of the patient at the age of 5 years and her parents confirmed paternity and identified a novel de novo *SMARCA2* missense mutation c.3721C>G; p.Gln1241Glu (ENST00000349721 NM_0030703), confirmed by Sanger sequencing in the patient. (See Fig. 1a and b for IGV plot and Sanger sequencing chromatogram, respectively). This novel variant is highly conserved and not reported in 1000G, ExAC, or EVS. In the ExAC database, nonsynonymous missense, stopgain, and splice variants in exon 26 are very rare with MAF < 0.0001 and one splice variant with MAF 0.001–0.0001. Annotation from insilico prediction tools was SIFT 0.184 (tolerated), PolyPhen2 0.900 (possibly damaging), mutation taster 0.999 (disease causing), and CADD 22.5 (top 1% most deleterious).

**Figure 2:**
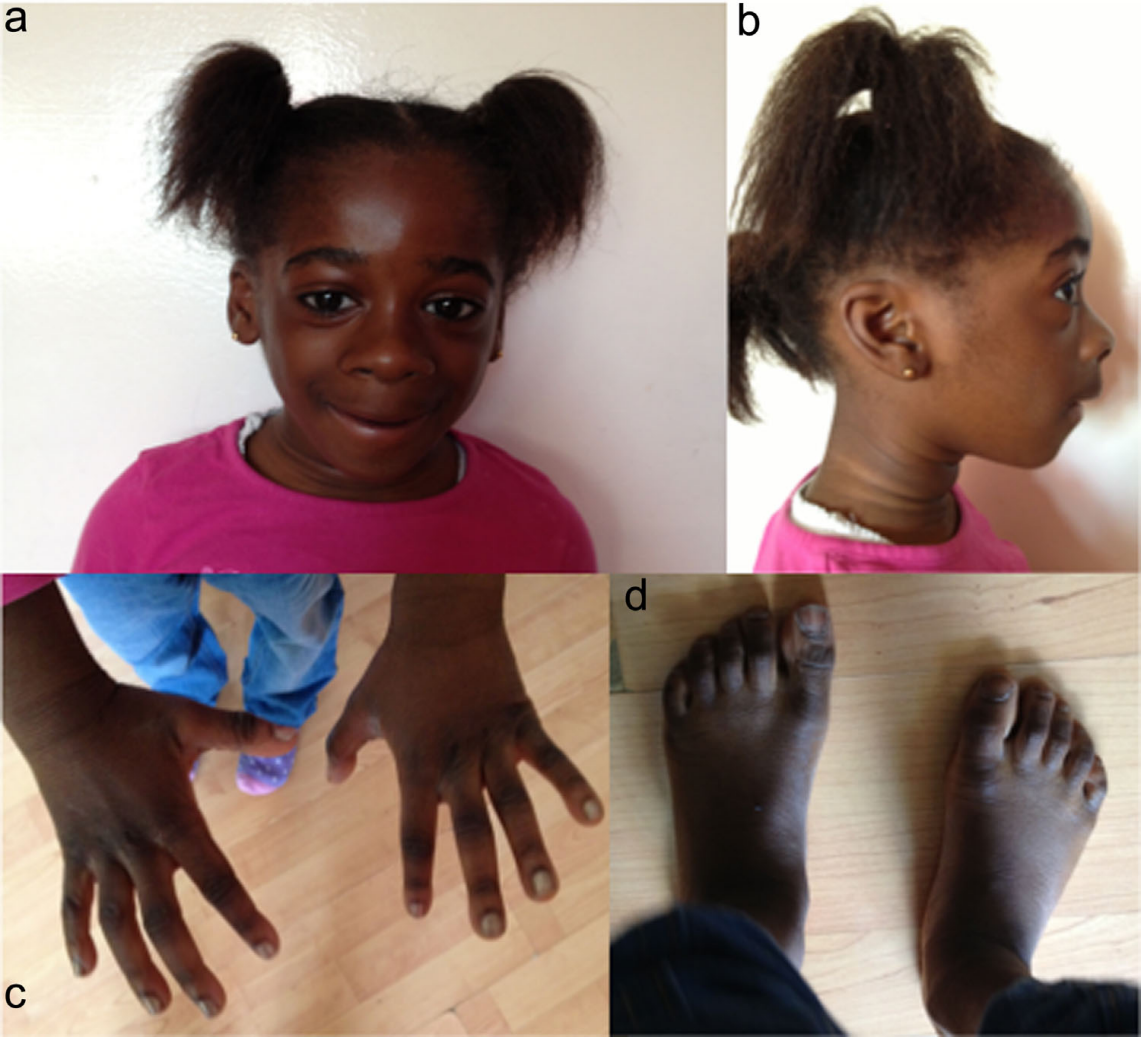
**a** and **b**: Clinical photographs demonstrating features of skin wrinkling, frontal balding, broad nasal base, upturned nasal tip, thick alae nasi, broad and long philtrum, thin upper vermillion, and thick lower vermillion, anterior projection of the upper lip over the premaxilla. **c**: Clinical photographs of hands with slightly thick distal phalanges. **d**: Clinical photograph of feet. [Color figure can be viewed at wileyonlinelibrary.com].

Following the exome sequencing findings, we reviewed her clinical examination at 5 years 5 months and observed skin wrinkling, frontal balding, a broad and long philtrum, broad nasal base, upturned nasal tip, thick alae nasi, anterior projection of the upper lip over the premaxilla, and thin upper vermillion and thick lower vermillion border. She had slightly thick distal phalanges in the hands. (See Fig. 2a–d for clinical photographs). She had eczema particularly involving the elbows and knees. Her clinical phenotype of specific craniofacial features, reduced speech, intellectual disability, autism, and seizures along with a de novo *SMARCA2* mutation was consistent with the Nicolaides–Baraitser syndrome spectrum (NCBRS) [Sousa and Hennekam, 2014].

The institutional review boards and ethical committees of King's Health Partners approved the study. The participants gave written informed consent in accordance with the Declaration of Helsinki.

## DISCUSSION

We report a patient with MAE and features of NCBRS. Although seizures are reported in two‐thirds of patients with NCBRS, neither seizure semiology nor specific epilepsy syndromes have been well described. The patient described in this study was diagnosed with MAE syndrome and the diagnosis of NCBRS in this patient was only made following identification of the *SMARCA2* mutation and clinical re‐examination. This was because the NCBRS phenotype is progressive and in younger ages the features are subtle and difficult to recognize. Moreover, there is no reported association between MAE and NCBRS. A specific role for *SMARCA2* mutations has been implicated in NCBRS with at least 80% of patients carrying a mutation in *SMARCA2* (OMIM 601358) [Van Houdt et al., 2012], but to our knowledge all causative mutations have been located in exons 15–25, and never before in exon 26. *SMARCA2* is a chromatin remodeling gene, and mutations in another gene involved in this pathway *CHD2* are known to cause MAE, suggesting this pathway as a potential therapeutic target [Carvill et al., 2013].

There are no reported specific seizure phenotypes or EEG abnormalities for NCBRS, although epilepsy occurs in two‐thirds of patients. In a series of 61 individuals with NCBRS, the median age at the first seizure was 18 months [Sousa and Hennekam, 2014]. Additionally, as in our patient, a co‐occurrence of decreasing mental abilities with the onset of seizures (epileptic encephalopathy) in NCBRS has been observed [Sousa and Hennekam, 2014]. Epileptic encephalopathy is also frequent in MAE, and it is possible that the co‐existence of NCBRS /MAE is more common than expected.

**Figure 3:**
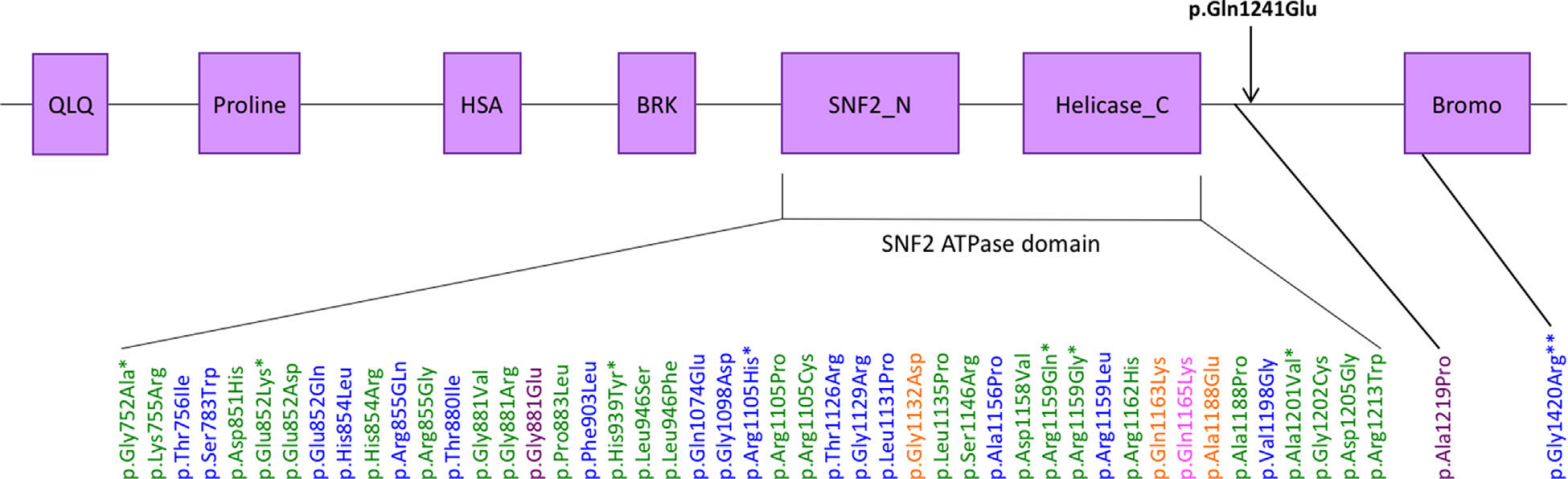
Schematic diagram of SMARCA2 protein and its domains and location of missense mutations associated with NCBRS. QLQ, glutamine‐leucine‐glutamine domain; Proline, proline‐rich domain; HSA, small helicase/SANT associated domain; BRK, brahma and kismet domain; SNF2_N and Helicase_C; Bromo, acetyl‐lysine binding bromodomain. Patient's mutation in black. Mutations reported first by [Van Houdt et al., 2012] in green, [Wolff et al., 2012] in orange, [Sousa and Hennekam, 2014] in blue, [Bramswig et al., 2015] in purple, and [Ejaz et al., 2016] in pink. *Recurrent mutations. **Patient without classical NCBRS but with overlap features of intellectual disability, absent speech and seizures. [Color figure can be viewed at wileyonlinelibrary.com].

*SMARCA2* is located on chromosome 9p24.3 and its longest transcript has 34 exons. Thus far, 62 missense mutations and three in‐frame deletions clustering in the ATPase domains of exons 15–25 of *SMARCA2* have been reported in patients with NCBRS [Sousa and Hennekam, 2014; Bramswig et al., 2015; Ejaz et al., 2016] (see Fig. 3 for schematic diagram of SMARCA2 protein and location of mutations). These mutations are thought to have a dominant negative effect by abolishing the ATP hydrolyzing engine potential of the protein, removing the ability of the protein to reposition histones on DNA. Recently, one other patient with NCBRS but no seizures has been reported to carry a mutation c.3655G>C, pAla1219Pro, located outside but close to the ATPase domain, [Bramswig et al., 2015]. To our knowledge, the patient reported here carries the first mutation located in exon 26, also in close proximity of the ATPase domain. We hypothesize that both mutations similarly affect ATP hydrolysis and chromatin remodeling ability. Sousa and Hennekam [2014] reported a case with a mutation in the Bromo domain, in exon 30 (p.Gly1420Arg). Their case did not have the typical facial features seen in NCBRS but had overlap features of severe intellectual disability, absent speech and seizures. The absence of mutations reported outside exons 15–25 may be due to restricted diagnostic laboratory sequencing excluding other areas of the gene based on previous association [Sousa and Hennekam, 2014]. Based on our findings, we would suggest incorporating exon 26 in clinical laboratory testing.

Genetic aberrations involving exon 26 in addition to ATPase disruption have also been described. NCBRS has been reported with a 32 kb de novo in‐frame *SMARCA2* deletion affecting exons 20–26 [Wolff et al., 2012] and a 55 kb interstitial deletion involving exons 20–27 was reported in a patient with the phenotypically similar Coffin–Siris syndrome [Tsurusaki et al., 2014]. These patients had epilepsy, but the associated seizure semiology and epilepsy syndromes merit further description.

*SMARCA2* is one of the six genes that encodes the catalytic subunit components of the SWItch/sucrose nonfermentable like chromatin‐remodeling complex (SWI/SNF complex). The SWI/SNF complex is evolutionarily highly conserved from yeast to humans and works as a chromatin remodeler, altering chromatin structure through ATP hydrolysis. SWI/SNF proteins regulate gene expression by re‐positioning nucleosomes and altering DNA transcription. A close connection between the SWI/SNF complex and neurological development has been implicated through the identification of numerous mutations in genes encoding the subunits in a range of neurodevelopmental disorders such as Coffin–Siris syndrome, sporadic intellectual disability, autism spectrum disorder, schizophrenia, and Kleefstra syndrome [Son and Crabtree, 2014].

We conclude that (i) NBCRS spectrum features can be caused by mutations outside the ATPase region of *SMARCA2*; and that (ii) the epilepsy phenotype in NBCRS can be consistent with MAE syndrome. This study underlines the fact that, in rare genetically heterogeneous conditions like MAE, where the patient is primarily under the care of a non‐clinical specialist geneticist, exome sequencing can help aid in making clinical syndromic diagnosis.

## INTERNET SOURCES

CADD http://cadd.gs.washington.edu

Mutation Taster http://www.mutationtaster.org

PolyPhen‐2 http://genetics.bwh.harvard.edu/pph2

SIFT http://sift.jcvi.org

## Supporting information

Additional supporting information may be found in the online version of this article at the publisher's web‐site.

**Figure 1**. EEG of patient performed at 17 months demonstrating frequent generalized bursts of polyspike and wave activity during wakefulness against a normal background.

**Figure 2**. EEG of patient at 17 months demonstrating electrographic correlate of negative axial myoclonus with a decrease in EMG signal correlating with an abrupt loss of posture.
