# Supplementary figures and images for: New SMARCA2 mutation in a patient with Nicolaides–Baraitser syndrome and myoclonic astatic epilepsy
Source: Am J Med Genet A. 2016 Sep 26;173(1):195–9. doi: 10.1002/ajmg.a.37935 (PMC5516167; doi:10.1002/ajmg.a.37935)

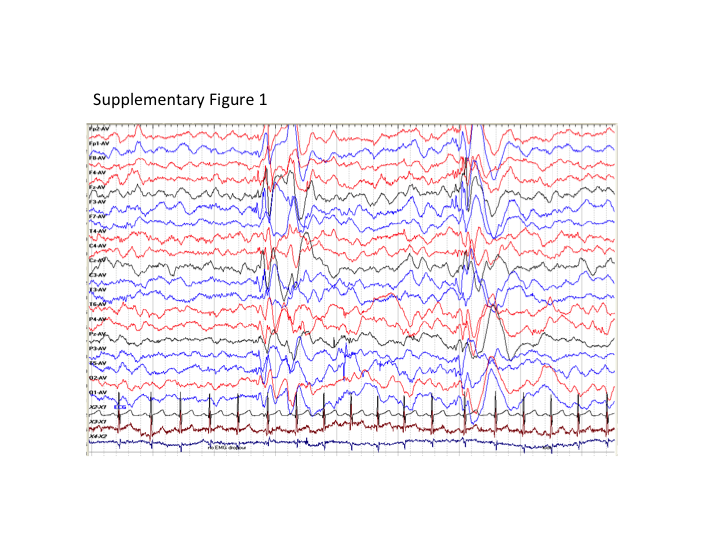

Supplement: Supplementary file 1 — Figure 1. EEG of patient performed at 17 months demonstrating frequent generalized bursts of polyspike and wave activity during wakefulness against a normal background. [file AJMG-173-195-s001.tif]

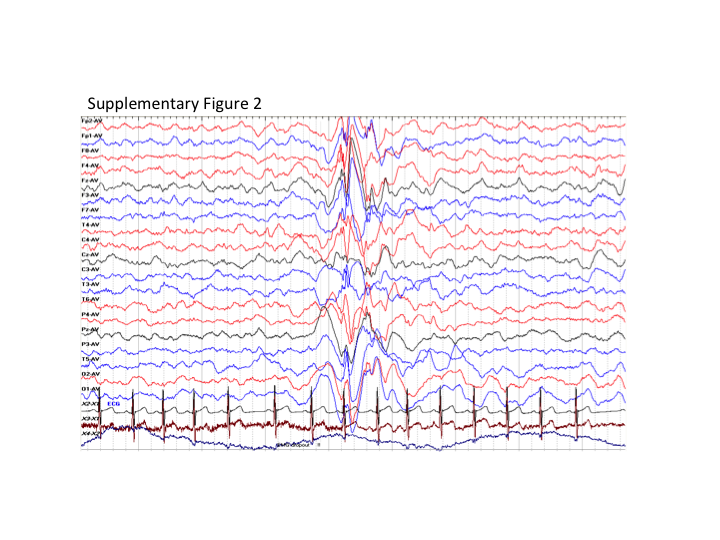

Supplement: Supplementary file 2 — Figure 2. EEG of patient at 17 months demonstrating electrographic correlate of negative axial myoclonus with a decrease in EMG signal correlating with an abrupt loss of posture. [file AJMG-173-195-s002.tif]
